# What Matters Most to Lung Cancer Patients? A Qualitative Study in Italy and Belgium to Investigate Patient Preferences

**DOI:** 10.3389/fphar.2021.602112

**Published:** 2021-03-04

**Authors:** Serena Petrocchi, Rosanne Janssens, Serena Oliveri, Reinhard Arnou, Ilaria Durosini, Paolo Guiddi, Evelyne Louis, Marie Vandevelde, Kristiaan Nackaerts, Meredith Y. Smith, Giulia Galli, Filippo de Marinis, Letizia Gianoncelli, Gabriella Pravettoni, Isabelle Huys

**Affiliations:** ^1^ Applied Research Division for Cognitive and Psychological Science, IEO, European Institute of Oncology IRCCS, Milan, Italy; ^2^ Department of Pharmaceutical and Pharmacological Sciences, KU Leuven, Leuven, Belgium; ^3^ Department of Pulmonology/Respiratory Oncology, University Hospital Leuven, Leuven, Belgium; ^4^ Alexion Pharmaceuticals, Boston, MA, United States, University of Southern California School of Pharmacy, Los Angeles, CA, United States; ^5^ Department of Medical Oncology, Fondazione IRCCS Istituto Nazionale dei Tumori, Milan, Italy; ^6^ Thoracic Oncology Division, IEO, European Institute of Oncology IRCCS, Milan, Italy; ^7^ Department of Oncology and Hemato-Oncology, University of Milan, Milan, Italy

**Keywords:** patient preferences, drug decision-making, drug development, patient-centered research, lung cancer, benefit-risk assessment, focus group discussions

## Abstract

**Background: **The potential value of patient preference studies has been recognized in clinical individual treatment decision-making between clinicians and patients, as well as in upstream drug decision-making. Drug developers, regulators, reimbursement and Health Technology Assessment (HTA) bodies are exploring how the use of patient preference studies could inform drug development, regulatory benefit risk-assessment and reimbursement decisions respectively. Understanding patient preferences may be especially valuable in decisions regarding Non-Small Cell Lung Cancer (NSCLC) treatment options, where a variety of treatment options with different characteristics raise uncertainty about which features are most important to NSCLC patients. As part of the Innovative Medicines Initiative PREFER project, this qualitative study aimed to identify patient-relevant lung cancer treatment characteristics.

**Methods: **This study consisted of a scoping literature review and four focus group discussions, 2 in Italy and 2 in Belgium, with a total of 24 NSCLC patients (Stages III-IV). The focus group discussions sought to identify which treatment characteristics patients find most relevant. The discussions were analyzed thematically using a thematic inductive analysis.

**Results: **Patients highlighted themes reflecting: 1) positive effects or expected gains from treatment such as greater life expectancy and maintenance of daily functioning, 2) negative effects or adverse events related to therapy that negatively impact patients’ daily functioning such as fatigue and 3) uncertainty regarding the duration and type of treatment effects. These overarching themes were consistent among patients from Belgium and Italy, suggesting that treatment aspects related to efficacy and safety as well as the psychological impact of lung cancer treatment are common areas of concern for patients, regardless of cultural background or country.

**Discussion: **Our findings illustrate the value of using qualitative methods with patients to identify preferred treatment characteristics for advanced lung cancer. These could inform a subsequent quantitative preference survey that assesses patient trade-offs regarding treatment options.

## Introduction

Non-Small Cell Lung Cancer (NSCLC) represents approximately 85% of the lung cancer forms worldwide (Jemal et al., 2011). Traditional medical treatments for advanced stages of NSCLC consist of a combination of chemotherapy and/or targeted therapy (Zappa and Mousa, 2016; Planchard et al., 2018; Hanna et al., 2020). Recently, new NSCLC treatments have been introduced, such as immunotherapy, the combination of immunotherapy and chemotherapy, and local stereotactic body radiotherapy (Ellis and Vandermeer, 2011; Aumann et al., 2016; Novello et al., 2016; Reck et al., 2016; Planchard et al., 2018; Shafique and Tanvetyanon, 2019; Hanna et al., 2020). Different treatments have both different benefits in terms of progression-free survival, overall survival and objective response rate and different risks or side-effects. For example, frequent side-effects of chemo-immunotherapy are weight increase, hair loss, pain, nausea, vomiting, breathing problems and fatigue. All of these may negatively impact patients’ body image perception (Bahrami et al., 2017) and health-related quality of life (Blinman et al., 2010; Grassi et al., 2017).

This variety of lung cancer treatment options (chemotherapy, immunotherapy, chemotherapy-immunotherapy, targeted therapy), their benefits (e.g., progression-free survival, overall survival, response rate), and risks (e.g., fatigue, negative body perception) underscore the need for informed clinical treatment decision-making that takes into consideration patients’ preferences^1^. Such decisions require patients and their clinicians to trade-off between more aggressive treatments, with a more negative impact on their quality of life, and alternatives that may be less effective but which carry fewer adverse events, and hence less negatively affect health-related quality of life (Blinman et al., 2010; Hajjaj et al., 2010).

In view of this large variety of NSCLC treatment options that are associated with a range of different characteristics, decisions concerning lung cancer treatments may be classified as “preference sensitive” (Lillie et al., 2014). In such decisions, the “right” decision depends the value that patients place on particular health outcomes. The potential value of patient preferences has not only been recognized in the clinical individual (“micro”) decision-making context, but also in upstream (“macro”) drug decision-making. Drug developers, regulators, reimbursement and Health Technology Assessment (HTA) bodies are exploring how the use of patient preferences could improve drug development, regulatory benefit risk-assessment and reimbursement decisions respectively (Cook et al., 2019; Hines et al., 2020; PPI, 2019).

Patient preferences can be investigated through patient preference studies via qualitative methods (e.g., interviews, focus group discussions) and/or quantitative methods (surveys using specific preference elicitation techniques, such as discrete choice experiment or swing weighting) (Van Overbeeke et al., 2019). Whereas qualitative methods are generally exploratory (see for example Bailo et al., (2019)), quantitative methods often require patients to choose between treatment alternatives and provide quantitative preference evidence (PPI, 2019). Researchers have stressed the importance of qualitative methods for investigating patient preferences; qualitative methods have been described to generate richer information than quantitative methods as they permit sensitive topics to be discussed (Coast et al., 2012; Hollin et al., 2020).

Several patient preference studies have been performed among lung cancer patients with three systematic reviews summarizing a portion of this. A first systematic review by Blinman et al., (2010) summarizes five studies between 1997 and 2009 on the subject of survival benefits that NSCLC patients expect to make the chemotherapeutic toxicity worthwhile. However, the emphasis on chemotherapy makes it difficult to generalize the findings to other therapies. In addition, the studies included in this systematic review were conducted before newer treatments such as immunotherapy were available, which may affect patients’ preferences. Furthermore, the studies were all quantitative, which suggests that the attributes included in these studies were not selected by patients, but rather by clinicians and researchers. This absence of qualitative research for attribute development also contrasts with the recent recommendation of Hollin et al., (2020) and Coast et al., (2012) to use qualitative research for developing attributes included in subsequent preference surveys. Schmidt et al., (2016) conducted a more recent systematic review, which includes 17 studies between 2000 and 2012. The scope of this review was broader than the one of Blinman et al.,; however, the majority of the included studies focused on chemotherapy and had a quantitative approach. Another recent systematic review (Sugitani et al., (2020)) found 15 studies published from 2000 to 2020, nine of them specifically on lung cancer patients. Finally, another systematic review found that health literacy, numeracy, and locus of control have an impact on health-related preferences and decisions (Russo et al., 2019). The included studies considered chemotherapy and surgery or radiation, and results from the systematic review suggested that, according to patients, health-related quality of life and overall survival were the most important features of a therapy. Besides these three systematic reviews, other patient preference studies have been conducted with conflicting results. This could be attributable to the fact that the scopes of these studies differ greatly. Most of them have an emphasis on a particular goal, such as a specific patient group (Hirose et al., 2005; Hirose et al., 2009; Schmidt et al., 2017), specific treatment (Peeters et al., 2012) or specific interest in characteristics such as emotional wellbeing (Mosher et al., 2017). In contrast, the present qualitative study aimed to identify patient-relevant lung cancer treatment characteristics across different therapies (including newer types of therapies such as immunotherapies) according to advanced lung cancer patients.

## Materials and Methods

### Study Context

This study was conducted as part of the *Patient Preferences in Benefit-Risk Assessments during the Drug Life Cycle* (PREFER) project. PREFER aims to develop evidence-based recommendations to guide industry, regulatory authorities, and HTA bodies (including reimbursement agencies and payers) on how and when patient preference studies should be performed and when the results can be assessed and used in order to support and inform medical product decision-making (“Including the patient perspective”, 2020).

### Qualitative Approach, Data Collection Methods and Sources

This study aimed to identify patient-relevant lung cancer treatment characteristics. The qualitative study design involved a scoping literature review and focus group discussions with patients (Durosini et al., 2021). The literature review informed the list of treatment characteristics used in the subsequent focus group discussions, in which patients were first asked openly about which treatment characteristics (such as potential improvements and side-effects) matter most to them, and afterwards reflect on examples of treatment characteristics retrieved via the literature search. (Supplementary Appendix SA). The literature review extracted treatment characteristics from: 1) previously performed preference studies among lung cancer patients, 2) benefits and risks of treatments already being prescribed to lung cancer patients, and 3) treatment characteristics of medicines that are currently being studied in clinical trials for the treatment of lung cancer patients. Searches involved two electronic databases (Web of Science and PubMed) and used free text terms and Medical Subject Headings (MeSH). Variations of the following key search terms were adopted: “Lung Cancer” AND “Patient preferences”. Only research papers published in English were considered. Both reviews and meta-analyses including original articles were evaluated for inclusion in the scoping review. The results were screened using a twofold process. First, the title and abstract were screened based on the inclusion criteria that the studies had to: 1) assess the treatment of lung cancer and 2) assess patient preferences for lung cancer treatments. Afterwards, the full text of the selected article were reviewed to ensure that the article was relevant to the scoping review based on the above inclusion criteria. If the article met the inclusion criteria, it was included in the review and information from the study was extracted for analysis. Based on the review, a final table including both positive aspects (improvements) and side-effects related to cancer therapies was developed (See Supplementary Appendix SA).

The focus group discussions sought to identify which treatment characteristics patients found most relevant and why Supplementary Appendix SC). Focus group discussions were selected as the method for data collection because they allow for interactivity between participants, active discussions guided by the researchers, and thereby may generate topics that researchers were previously unaware of (see Durosini et al., 2021).

### Sampling Strategy and Process

Advanced stage lung cancer patients were included because they often have received different kinds of treatments and are thus able to reflect on a broad range of different characteristics from different therapies. Moreover, uncertainty on behalf of decision-makers (clinicians, patients, regulators, HTA/bodies and reimbursement agencies) seems to be particularly present in the context of advanced stage lung cancer. This is caused by the increasing amount of treatment options and treatment combinations for all stages of NSCLC, but for advanced stage lung cancer in particular as described in the introduction (see also Supplementary Appendix SB).

Participants were recruited by the treating oncologists at the Thoracic Oncology Division of the European Institute of Oncology in Milan and at the Respiratory Oncology Department of the KU Leuven University Hospital in Leuven.

Inclusion and exclusion criteria were defined and described in the protocol paper of the study (Durosini et al., 2021). A formal invitation letter was sent to those patients expressing interest in the study and who met the inclusion criteria. Those interested in participating were then contacted by telephone to plan the date and time of the focus group discussions. Ethical approval was obtained from both study centers before starting participants recruitment^2^ and participants signed a written informed consent before participation.

### Data Collection and Tools

From August 2019 to October 2019, participants were invited for the scheduled focus group discussions. At the beginning of each discussion, an informed consent process took place and a consent form was signed by each participant. The discussion consisted of the following steps (see Supplementary Appendix SC):

• Welcome time, provision of the information sheet and signing of the consent form;
• Introduction of the focus group discussion including an explanation of some basic discussion ‘rules’ such as the importance of listening to each other, the aims of the study;
• Introduction of the main topics of the discussion and time for participants to introduce themselves;
• Introduction and discussion on the first question/topic: *“when you undergo a treatment for lung cancer, what type of improvement do you expect from it? With improvement, we mean benefits or desirable effects. Why?”*;
• Introduction and explanation of examples of improvements from the literature review (see Supplementary Appendix SA) and time for participants to reflect on the list;
• Break;
• Introduction and discussion on the second question/topic: “*When you undergo a treatment for lung cancer, what type of side-effects would make you want to doubt whether you want to keep on taking the treatment? Why? What type of these side-effects would make you stop taking the treatment? Why?”*;
• Introduction and explanation of potential examples of side-effects from the literature review (see Supplementary Appendix SA) and time for participants to reflect on the list;
• Introduction and discussion on the third question/topic: *“What type of side-effects would make you want to reconsider whether you want to continue the treatment? What type of improvements would make you want to accept more of the side-effects we just talked about?”.*
• Introduction and discussion on the fourth question/topic: *“Are there any other aspects of lung cancer treatment, besides the different side-effects and improvements we just talked about that would influence your choice to take or stop taking a lung cancer treatment? Why?”*
• Summary, Conclusions and Final Greetings.

The full discussion guide can be retrieved in the Supplementary Appendix SC. During the discussion, the moderator probed about whether a specific point was found important across participants, whether there was consensus or disagreement on certain aspects. The overall duration of the discussions ranged from 1.5 to 2.5 h and the discussions were conducted by the authors of this paper (SP, SO, ID, PG in Italy and RJ, RA, IH in Belgium), who have experience with qualitative research approaches and conducting focus group discussions. The moderator facilitated the discussion, allowing participants to respond spontaneously to the issues raised. The discussions were conducted in person in a silent and comfortable room, and they were audio recorded with participants’ permission. To increase procedural comparability among the discussions conducted by the two different teams, both teams used the same discussion guide and weekly meetings between moderators and other members of the research team were organized before and after the discussions took place. In all discussions, participants were asked to complete a short questionnaire (Supplementary Appendix SD) to gather information on socio-demographics and health literacy via the Chew Brief Literacy scale (Chew et al., 2008).

As for the sample size, it is worth noting that data collection for qualitative research does not have clear guidelines on how much data should be included in a study (Brod et al., 2009). Several authors indicate that qualitative data should be collected until “data saturation”, which has been defined as the point when “no new information or themes are observed in the data”, when redundancy is reached in data analysis and signals to researchers that data collection may cease. Several studies found that data saturation can be achieved after conducting four to six focus groups (Morgan, 1996; Brod et al., 2009; Kerr et al., 2010; Hennink et al., 2019), especially when the goal is to identify “core” issues. Since the aim of the present study was indeed to identify “core” attributes among lung cancer patients with regard to their treatment, we estimated that four focus groups across two different countries (Italy and Belgium) would be sufficient to reach data saturation (see also Durosini et al., 2021) Following qualitative data collection, it appeared that the same themes were observed across the focus group discussions. Hence, it was decided that no additional data was needed.

### Data Processing and Data Analysis

U Mann-Whitney tests were carried out to investigate possible differences between Italian and Belgian participants in terms of stage of disease, treatment characteristics, socio-demographics, and health literacy. The audio-recordings were transcribed verbatim in the original language and then translated into English by a professional translation company. Transcripts and notes from the focus group discussions were thematically analyzed using an iterative approach as described in the framework method by Lacey and Luff (2009) (see Table 1) and using Nvivo v.11.

**TABLE 1:** Iterative steps of the framework method followed for the thematic analysis of the focus group discussions. The charting step (i.e., summarizing of the data based on the themes identified through the analysis), as described by Lacey and Luff (2009), was performed during the writing of this paper and is therefore not mentioned in the table.

1. Familiarization	SP, SO, RJ, and RA thoroughly read and re-read the transcripts. They used the margins of the transcripts to write down analytical notes, thoughts or impressions (e.g., when focus group participants expressed exceptionally strong or contrasting views)
2. Identifying a thematic framework	To identify an initial thematic framework, SP, SO, RJ, and RA independently coded the text, meaning that they attached specific themes or concepts to particular paragraphs, based on the research aims of the study. These codes were different factors such as treatment outcomes, side-effects and symptoms patients mentioned during the focus group discussions
3. Coding	SP, SO, RJ, and RA discussed these preliminary codes to assess whether they interpreted the focus group discussions in the same manner and to reach consensus about the final coding list. The final coding list (i.e. framework) consisted of the final list of characteristics, each with examples of what ideas or elements could be summarized under that code. NVivo (QSR international) was used to code the transcripts using the final coding list
4. Mapping and interpretation	Meetings were organized between SP, SO, RJ, and RA to discuss their coding. This process took into consideration the differences between the Italian and Belgian focus group discussions but also between the focus group discussions within one country

An inductive thematic analysis was conducted, allowing the transcripts to determine the themes. Four researchers, two in Italy and two in Belgium, independently coded all the transcripts. A first phase of familiarization involved reading the material, taking initial notes, and getting familiar with the data. Using Nvivo v.11, the second phase involved the data coding, highlighting sections of the text and labeling them with a short code to describe the content. The codes are those aspects that emerged from the discussions and were found to be relevant across group members. In the third phase, the codes were then grouped in themes, in which every theme is a combination of several codes. The final phase involved defining and naming the themes. Throughout the analysis, an iterative, constant comparative analysis approach was used to enable continuous modifications and extensions of the themes to ensure that all key aspects could be incorporated through these modifications. The lists of themes was then compared and combined across Italy and Belgium to generate a comprehensive list. During this phase, overarching themes were detected, whilst considering similarities or potential differences in the sub-themes identified.

## Results

### Literature Review

From the literature review, a list of examples of treatment characteristics included in the focus group discussions was derived (see Supplementary Appendix SA).

### Focus Group Discussions

#### Participants’ Characteristics

Twenty-four advanced stage lung cancer patients participated in the focus group discussions (age range: 42–78, Mage = 61, SDage = 8.5; 62% men), equally distributed in Belgian (*n* = 12) and Italian (*n* = 12) discussions (6 patients in each discussion). Mean age of patients at the time of diagnosis was 58 years (*SD* = 8; Age range: 41–73). Response rates ranged between 50% and 57% in Italy and Belgium and across the four focus group discussions. Reasons for refusal to participate were: 1) extreme psychological suffering due to NSCLC diagnosis, 2) painful physical symptoms, 3) long distance from the hospital, 4) too little time available, 5) not interested and 6) preference to stay at home whenever they were not necessarily expected to be in the hospital (for quality of life). The majority of participants had a high-school degree (41.6%), whereas 37.5% did not finish the High School, and 20.8% had a university degree. U Mann-Whitney tests indicated that Belgian participants were significantly older than Italian participants (*U* = 111.5, *p* = 0.020), and significantly longer diagnosed with lung cancer than Italian participants (*U* = 109, *p* = 0.039). Gender and education distributions were not significantly different in the two groups of participants. Comparing Belgian and Italian patients, there was no difference even in the number of participants who were on treatment. Additionally, U Mann-Whitney tests revealed a significant difference between Italian and Belgian participants on the first question of the literacy scale (“How often do you have someone, like a family member, friend, hospital/clinic worker, or caregiver, help you read hospital materials?” retrieved from Chew et al., (2008)) (*U* = 108.5, *p* = 0.025) with Italian participants relying more on family members than the Belgian patients. There were no differences between the two groups on the other two questions included in the Chew Brief Literacy scale. Table 2 reports all participants’ characteristics.

**TABLE 2:** Participants’ characteristics distinguished by country. LC = lung cancer.

	Italy	Belgium
**Age**
M (sd) - range	57.33 (8.56)–42–72	64.92 (6.82)–52–78
**Gender**
Males Females	41.6% 58%	83.3% 16.7%
**Education**
Did not finish high school High school Diploma Higher education or university degree	58% 41.6% -	16.7% 41.7% 41.7%
**Currently on LC treatment**
No treatment or only symptomatic treatment Immunotherapy Chemotherapy Chemotherapy and immunotherapy Biological therapy	- 33.3% 16.7% 16.7% 33.3%	33,3% 41.7% 16.7% 8.3% -
**Number of treatment lines**
M (sd) - range	2.42 (3.6)–0–12	2.33 (1.44)–0–5
**Health literacy** - M (sd)
Item 1 Item 2 Item 3	2.58 (1.8) 3.83 (1) 3.67 (0.77)	4.33 (0.89) 3.92 (0.79) 3.83 (0.58)

#### Qualitative Results From Thematic Analysis

A thematic analysis was conducted on the focus group discussion transcripts. Data revealed differences in the specific side-effects experienced by NSCLC patients. More specifically, episodes of diarrhea, vomiting, and sexual impotence emerged in Italian focus group discussions, while they were not raised during the discussions in Belgium. In addition, the importance patients gave to specific side-effects (e.g. hair loss, cognitive limitations) varied between individual patients. However, there were apparent overarching themes of treatment characteristics where patients agreed upon during the discussion. In particular, three overarching themes emerged from the thematic analysis: 1) positive effects or expected gains from treatment, 2) negative effects or adverse events that patients want to avoid, and 3) uncertainty regarding the duration and type of treatment effects. These themes are described below.

##### Positive Effects or Expected Gains From Treatment

The first theme emerging in the four focus group discussions consisted of positive treatment effects such as greater life expectancy, decrease in cancer growth, cancer remission, and maintenance of daily functioning. Patients reported that one important reason to prefer a particular treatment is that it increases their life expectancy and prolongs the patient’s life. Another important feature for patients was that the treatment ensures that the cancer grows less rapidly or stops growing.

Cancer remission was another important feature of the treatment. Patients expected to see their cancer stop growing, and reduce in size. Lastly, participants highlighted the importance of being able to perform their daily activities. Participants stated they wanted to live as much as possible a normal life, and be able to continue their activities like before their cancer. In Table 3, we report on several quotes related to this theme.

**TABLE 3:** Examples of qualitative evidence of the themes.

Overarching Themes	Themes	Italy	Belgium
Positive effects or expected gains from treatment	Greater life expectancy	P6-Focus 1: “Oh, [I expect] that he makes me live a little more” P4-Focus 1: “It would be enough for me to know that it [the cancer] is blocked there. I've been fine so far and sometimes I don't realize I'm doing this So it would be enough for me to stop it.” P3-Focus 1: “Live as long as possible! When they say you have cancer it is not like when you break an arm that you say “heal”.”	P5-Focus 1: “I hope that the treatment prolongs my life so that I can see my grandchildren grow up” P2-Focus 1: “If you feel bad one day That is nothing in comparison to living a day longer” P5-Focus 1: “P5: Well, I’ve always continued to move forward. I even started sorting out my funeral two months ago. Hoping of course, that I can stay alive for a long time, and so that my wife does not have any problems later on. that everything is arranged and that she can be at ease. So that is how rational I am and yes I just carry on with my life.”
	Decrease in cancer growth	P6-Focus 1: “I expect that maybe it [the cancer] regresses a little and that he stays there as it is.”	P1- Focus 1: “Healing will not be possible, so limiting the progression is our goal” P1- Focus 2: “For most people, it is already too late to be able to cure them, all you can do at that point is reduce it”
	Cancer remission	P1-Focus 1: “I expect healing” P2-Focus 2: “I immediately tell you loud and clear: to heal. Which unfortunately did not happen. Do not do more treatments and do nothing. To heal”	P2-Focus 1: “The focus must always remain on the primary - on healing, and the rest is nice to have” P4-Focus 1 “If they say: ‘we are going to give you chemo and radiation’. ‘Yeah, go ahead’. What am I supposed to do? 1/3 chance of success, of healing”
	Maintenance of daily functioning	P3-Focus 1:“Try to live my life as normal as possible, as similar as possible to the previous one” P3-Focus 2:“It's not just a matter of not wanting to die, it's also a question of how you want to live, rather than not wanting to die” P5 -Focus 2: “P5: I can’t even ride a bicycle, imagine that. It is not that I can’t ride it, it is that I can’t get on it”	P5-Focus 2: “I think it depends on how livable your side-effects are, how enjoyable your life then still is” P2-Focus 1: “Being a bit more active than I am now. Sitting about at home isn’t easy if you’ve always worked. I mean it’s really driving me crazy” P2-Focus 1: “I would stop the treatment if my quality of life fell so low that it made me say: ‘why keep going?”
Uncertainty regarding the duration and type of treatment effects		P3-Focus 2: “Beforehand it lasted 1 h or 1 h and 10 min. Now I see that things have changed the initial phases were analysis, study and now the protocols have changed a bit... Beforehand I was doing it in 1 h and 10 min, now in 40 min. I am also used to it, I did it today. I am also used to it, apart from the first 24 h which” P1_Focus 2: “but the path is a bit long, you don’t recover straight away it takes a bit of time, it takes some years, at least that is the case in the experiences that I have encountered up until now”	P6-Focus 1: “It’s so new and long term, will it keep working, will it stop?” P7-Focus 1: “I had to wait 5 months for the results of a scan. That’s too long for me there’s too much uncertainty” P2- Focus 1: “If they would have told me beforehand: ‘Sir, your sense of taste will change and nothing will be tasty anymore Then you can say: ‘That is the way it has to be So that you know what you are up against” P1- Focus 2: “What could be the cause, what are the remedies, what are the chances of survival, what stages. there is unfortunately very little to be found, let's say at the Benelux level, of information. I have been lucky from day one to have had a very good communication, in my case with the professor” (treating physician)
Negative effects or expected loss from treatment	Severity of skin problems	P2-Focus 2: “Every day. One pill that I take every day. I find that this pill, compared to the other one, the other one really killed me. In the first few months I lost all of the skin from my hands, face, spots”	P7-Focus 1: “That’s a side-effect of the immunotherapy; it starts to itch, then I began to scratch it, but you keep scratching until you start bleeding”
	Severity of nausea	P1-Focus 1: I haven’t had any side-effects, apart from in January when I had chemo, nausea at the thought of eating fish, but zero side-effects	P2-Focus 1: “the misery that comes with it; being sick, feeling nauseous”
	Severity of diarrhea	P2-Focus 2: “The first treatment that I had, I had diarrhoea problems” P2 -Focus 2: “Better to die and then I was unless I had stomach sickness, diarrhoea, spots all over, blood coming out of here especially”	No example of this theme emerged in the Belgian focus groups
	Severity of vomiting	P2-Focus 1: “They brought me to accident and emergency because I could not eat any more, I vomited twenty times, after two times I had to stop it because it was highly” P2-Focus 1: “Yes, I couldn’t even manage to take Plasil, the side-effect was fainting, or almost.. it was vomiting, continuing and continuing and nothing ever came up because I wasn’t able to hydrate myself”	No example of this theme emerged in the Belgian focus groups
	Severity of hair loss	P3-Focus 1: “Yes I the history of hair, who loses them all on a personal level is devastating, okay you put on the wig but maybe if you could create something that controls this or that stimulates growth”	P5-Focus 1: “Losing my hair is the least of concerns” P2-Focus 1: “If I would go completely bald. I’m almost certain that that is something I absolutely cannot live with. Then I’d say ‘I stop”
	Severity of infections	P2-Focus 1: “I practically just eat fish, fruit, chicken. I have eliminated almost everything, pasta they tell me that I have a low immune system”	P2-Focus 1: “My daughter says ‘your immune system is at its lowest now. Don’t stand near ill people in the shop, stay away from the bakery, because if there is one person that is ill, then you will get ill too’ (…) you only have to catch a disease once and your treatment is completely ruined”
	Severity of infusion reactions	P3-Focus 2: “Look, because among those that I see thrombocytopenia colitis nausea reaction to the infusion”	P1-Focus 1: “The very first time, I had an internal reaction to the therapy. So I was shivering because I was so cold, then fever, forty degrees, and they were panicking at home saying: ‘you have to go to the hospital”
	Gravity of the edema	P3-Focus 1: “One thing that I would find hard to accept is too much swelling”	P1-Focus 2: “Those first two years, I didn’t have any (side-effects) apart from my edema but I could live with that just fine”
	Gravity of fatigue	P6-Focus 2: “Yes, I get tired easily” P6-Focus 1: “With chemo I was so tired, like that lady, I have always walked, I have climbed so many stairs and steps in my life and, conversely, since I have had chemo I have found it difficult to walk, to cook”	P7-Focus 1: “The only thing that I actually have, since I get that immunotherapy, yes tired, tired, tired, tired, I am always tired. I could always sleep. It is unbelievable”
	Gravity of weight fluctuations	P5-Focus 1: “I would not accept 120 kg because this weight gain is so frustrating for me that it really destroys me. I got to the point of not going out”	P1-Focus 1: “I’ve gained 20 kilos and I really don’t feel good about it If I want to go out, then it is like: ‘is my tummy not too big?’ And then I try to wear clothing that fit me as loosely as possible so that I can be a bit more comfortable.”
	Probability of renal failure	P3-Focus 2: “This is to say that if we stay on the topic of the side-effects, if we have therapy which makes the cancer chronic but creates additional new illnesses such as kidney failure”	P1-Focus 1“Then you get your test results and then you see that something about the kidney function is slightly less and that is - there were too few white blood cells in the urine”
	Probability of sexual impotence	P3-Focus 2: “P3: For me, as a man, impotence would certainly be of a different weight compared to the tube tube all my life but this, why? Because certain psychological problems of dissatisfaction would be triggered for a man; impotence and the impossibility of working are almost in the collective imagination.”	No example of this theme emerged in the Belgian focus groups
	Probability of having pain	P2-Focus 2: “I would do everything that I have done again, despite all the pain.”	P1-Focus 2: “There are an awful lot of kinds of lung cancer that give you really bad pain, where not even morphine is enough for the pain - yes and that’s something.”
	Probability of having hearing impairment	P4-Focus 2: “Well, I don’t know How can I say that? Besides, on the same day I lost my hearing, I don’t know if it was also linked to the first drug that I took I don’t know.”	P3-Focus 1: “But that was the decision: did I want to go deaf or did I want - well yes, that wasn’t an option for me”
	Probability of having weeping eyes	No example of this theme emerged in the Italian focus groups	P5-Focus 1: “The only nasty side-effect I have are tears and a runny nose. I eat with my fork in my right hand and I always need to have a tissue in my left hand”
	Probability of having cognitive limitations	P1 – Focus 2: It has really been more of a mental illness than a physical one P3-Focus 2: “Extreme conditions, you were talking about euthanasia and extreme conditions before, like in the case of the [name of a TV show] about [name of a well-known person in Italy who did euthanasia], that is an extreme condition, there is no point living like that.”	P3-Focus 2: “If they tell me I will get dementia, I will still continue to get treatment.” P1-Focus 2: “I will undergo every treatment as long as my brain functions.”
	Probability of having difficulty in breathing	P5-Focus 2: “My only fear was not breathing, the thing that I tried the two previous nights in hospital. But now I still have my breath. At the start I was doing three stairs and stopping with a fit, not now.”	P1-Focus 1: “but after an hour or an hour and a half - Then it’s like: ‘that’s enough for me, because you are so tired.”

##### Negative Effects or Adverse Events Related to Treatment

The second theme consists of categories related to common side-effects or adverse events of advanced stage lung cancer treatments (i.e., severe skin problems, nausea, hair loss, infections, and infusion reaction, gravity of edema, fatigue, and weight fluctuations) and categories related to less common side-effects (i.e., probability of renal failure, having pain, hearing impairment, cognitive limitations, difficulty in breathing). Table 2 shows examples of the discussions regarding those themes. Both Italian and Belgian participants stressed the importance of these effects because of their negative impact on patients' possibility to maintain their daily functioning or their psychological wellbeing. In contrast, diarrhea, vomiting, and sexual impotence emerging in Italian focus group discussions, were not raised by Belgian patients. Conversely, weeping eyes was only mentioned in the Belgian discussion.

##### Uncertainty Regarding the Duration and Type of Treatment Effects

The third theme relates to the uncertainty that patients experience regarding the long-term effects of their current treatment. Participants argued that both the uncertainty of the duration and type of negative effects and the uncertainty regarding the duration of positive effects were difficult for them to cope with. They stressed the role of patient-friendly but accurate treatment information and good communication with their healthcare providers as possible ways to help them cope with these uncertainties (Table 3).

## Discussion

This qualitative study identified patient-relevant characteristics of lung cancer treatment. The strenght of this qualitative study is the involvement of advanced stage lung cancer patients, who already experienced the main benefits vs. negative consequences of NSCLC therapies, including immunotherapy, chemotherapy, or a combination of both. For this reason, these persons can easily reflect and give their opinion on the outcomes of different therapies. The overarching themes reflecting treatment characteristics highlighted by patients in the focus group discussions were: 1) positive effects or expected gains from treatment such as greater life expectancy and maintenance of daily functioning, 2) negative effects or adverse events related to therapy that impact patients’ daily functioning and 3) uncertainty regarding the duration and type of treatment effects (Table 3). These overarching themes were consistent across patients from both Belgium and Italy, suggesting that treatment aspects related to efficacy and safety and the psychological effects of lung cancer and treatments are common areas of concern for advanced lung cancer patients, regardless of cultural background or country.

In contrast, differences regarding specific side-effects experienced by individual patients and how they had been experienced by individual patients were observed between participants within and across different focus group discussions in Italy and Belgium. More specifically, diarrhea, vomiting and sexual impotence were not raised by Belgian patients and weeping eyes was only mentioned in the Belgian discussion.

Although there is nothing in the published research literature to date suggesting that there are systematic differences between Italian and Belgian lung cancer patients concerning these side-effects, as with all qualitative research, it is possible that these differences were idiosyncratic to the specific group of patients who participated in the focus group discussions. In addition, it is possible that hitherto unstudied cultural differences in how side-effects and/or symptoms are experienced, acknowledged and expressed may account for these differences. For these reasons, conducting this study with other patients, and for example in additional countries, could lead to the identification of additional specific characteristics. Likewise, conducting this study when newer NSCLC treatment algorithms or newer (combinations of) medications become available could also lead to could also lead to the identification of additional treatment characteristics. Further research could assess the impact of treatment experience by setting-up a longitudinal design; conducting additional focus group discussion with the same patients in the beginning, in middle and at the end of their therapy to assess whether additional characteristics would emerge. Other patient and contextual factors that could influence their opinions and preferences are: 1) patients’ individual knowledge and need for information concerning therapies; some patients may want to learn about therapies whereas others not and the characteristics raised by these patients could differ between those groups of patients; 2) the way patients are supported and educated could also explain differences in opinions; e.g. participants with a supportive primary caregiver at home base might raise other aspects of the therapy vs. someone with difficult access to the hospital and/or a very small social network and 3) the time frame patients have (or need) to reflect on treatment decisions. It would therefore be interesting to further investigate heterogeneity in preferences and explain potential differences and the impact of these factors and patient characteristics on patient preferences. For example, it would be interesting to perform a further analysis of cross-country similarities and differences between Italian and Belgian patients and try to explain the impact of treatment experience on our findings. For the same reason, it would be interesting to apply the same methodology in additional countries to assess further if cultural aspects or other patient characteristics affect patient preferences. These are clearly important topics deserving further research in this regard, especially due to their implications for product manufacturers who are seeking to develop products, in most instances, for global markets, and regulatory and reimbursement decision-makers whose decisions impact multiple patients in one or more countries.

However, in view of limited evidence from lung cancer patient preferences regarding newer lung cancer therapies, we believe that this study may be informative for healthcare stakeholders interested in using patient preferences to inform the development, evaluation and prescription of lung cancer treatments. Firstly, lessons from applying this qualitative methodology may inform the development of PREFER’s evidence-based guidelines for future preference study developers and assessors on how to perform qualitative preference research that aims to inform decision-making across the drug life cycle. Secondly, the results may inform the development of a quantitative preference survey that elicits patients’ trade-offs for the characteristics of lung cancer treatment.

Thirdly, the identified treatment characteristics may inform healthcare stakeholders involved in the development and evaluation of lung cancer treatments (academia, clinicians, drug developers, patient organizations, regulators and HTA/reimbursement bodies) to understand the value of lung cancer treatment outcomes according to patients. More specifically, these characteristics may inform academia, patient organizations and drug developers on the design of patient-centered clinical trials, e.g. via the identification of clinical trial endpoints and patient-reported outcome measures beyond those that have traditionally been included in lung cancer treatment trials. For example, we identified that uncertainty and psychological aspects of lung cancer and lung cancer therapy are important to patients. It may therefore be appropriate to include a measure in clinical trials that assesses the psychological impact of the therapy on patients. In addition, evidence on how important patients find these different treatment outcomes may be used in marketing authorization and reimbursement (“macro”) decision-making. Patient preferences may add the available evidence on benefits and risks already considered during the decision process as well as complement existing decision criteria for marketing authorization and reimbursement.

Finally, in individual (micro) decision-making, the ethical and legal mandate for patient involvement in medical care is well-accepted as is the importance of patients expressing their preferences, and in engaging in informed choice in treatment decision-making (O’Connor et al., 1997; Say and Thomson, 2003). However, it has been highlighted that the preference elicitation process poses a challenge for doctors (Say and Thomson, 2003) in terms of time to spend to collect patients’ preferences and doctors’ abilities to elicit patients’ preferences. Our research may inform healthcare providers and clinicians of important factors on patients’ preferences, outside the traditional ones (e.g. age, performance status, comorbidities, histology and molecular pathology) (Novello et al., 2016). More specifically, these characteristics may be incorporated in decision aids that aim to improve shared decision-making between clinicians and patients as therapeutic decisions require value judgements of each of the treatments’ benefits and risks by doctors and patients combined together. An understanding of patients’ preferences could facilitate medical decision-making and promote more patient-centered health care.

## Limitations

Firstly, it is important to reflect on the representativeness of our sample. Care was taken to include different types of participants (with various disease and treatment histories, and inclusive of those patients in a relatively good condition as well as further progressed in the disease and who are typically harder to reach) to ensure that the eventually found characteristics are important to different types of patients. Notwithstanding, it is important to note that our results are not meant to be generalized to a population broader than the included sample and that they should not be viewed as representing the entire population from which the included patients were sampled, in this case, all stage III-IV NSCLC patients. As for any qualitative research, we also have to underline that we cannot make any conclusive statements regarding how the sample size may have affected the generalization of the results. However, we could speculate that the inclusion of additional patients and additional countries beyond the two included, could affect the derived themes. Secondly, the participants were selected by clinicians. This could be deemed as "cherry picking" certain types of patients that the clinician thinks would be most suitable for the study, e.g. those with the worst experiences, or those that are in a relatively good condition. Therefore, if only a certain type of patients was included, this could bias the outcomes of the study. However, as mentioned above, the clinicians took care to select different types of participants regarding current disease status and treatment history. Secondly, we found it useful to source patients with the clinicians' involvement, both because of their knowledge of the overall health status of the patients, and thus how to include different types of patients, as well as from a practical point of view. A last limitation of the study is the lack of a quantitative evaluation of the relevant themes. However, further quantitative research has been planned with the aim to complement and quantify the findings of the present qualitative study; the prioritized characteristics emerged from the present research will be ranked by patients using the Nominal Group Technique (Hiligsmann et al., 2013). In addition, a subsequent online survey has been planned to quantify the relevance of the treatment characteristics identified in this study, across a larger sample of lung cancer patients. This survey will also compare different quantitative methods for the elicitation and study of patient preferences.

## Conclusion

Our findings illustrate the value of using qualitative study methods with patients to identify preferred treatment characteristics for advanced lung cancer. These findings could inform a subsequent quantitative preference survey to assess patient trade-offs regarding treatment options. Previous preference research in this area, which relied predominantly on clinician-nominated treatment characteristics, used a comparatively limited set of characteristics that focused on survival and the severity of toxicity. In contrast, our preference study results indicate that patients consider a broader range of characteristics as being highly salient to their decision-making regarding cancer treatment options. Future research should examine whether our findings are transferable to other clinical settings.

The themes emerged from the present qualitative study may also inform: 1) drug developers on the design of patient-centered clinical trials and more specifically for the identification of clinical trial endpoints and patient-reported outcome measures, 2) regulators and HTA/reimbursement to understand whether the treatment being evaluated targets (clinical) outcomes that are relevant for patients and 3) healthcare providers when deciding on treatment options (e.g., via the development of decision aids that aim to improve shared decision-making between clinicians and patients).

## Data Availability

The data are not publicly available because they contain information that could compromise interviewees’ privacy and consent. Requests to access the datasets should be directed to serena.petrocchi@ieo.it.

## Footnotes

1
*The FDA refers to “patient preference information” as “qualitative or quantitative assessments of the relative desirability or acceptability to patients of specified alternatives or choices among outcomes or other attributes that differ among alternative health interventions” (* https://www.fda.gov/media/92593/download *). Attributes include types of benefits and risks associated with medical interventions. Attributes may also include other clinical and nonclinical aspects that can influence desirability or acceptability of medical interventions. (* https://www.fda.gov/about-fda/cdrh-patient-engagement/patient-preference-sensitive-areas-using-patient-preference-information-medical-device-evaluation *). In other words, patient preferences reveal what treatments or treatment characteristics patients find most important and how important they find them in comparison to each other*.

2 In Belgium, the “Ethische Commissie Onderzoek UZ/KU Leuven” approved the study (reference S63007). In Italy, the Ethical Committee of the European Institute of Oncology IRCCS IEO approved the study (reference 1027/19-IEO 1093)
