# Supplementary material for: What Matters Most to Lung Cancer Patients? A Qualitative Study in Italy and Belgium to Investigate Patient Preferences
Source: Front Pharmacol. 2021 Mar 4;12:602112. doi: 10.3389/fphar.2021.602112 (PMC7970036; doi:10.3389/fphar.2021.602112)
Supplement: Supplementary file 1 [file presentation1.pdf]

Supplementary Appendix

What Matters Most to Lung Cancer Patients? A Qualitative Study in Italy and Belgium to Investigate Patient Preferences

Serena Petrocchi*[1a], Rosanne Janssens*[2], Serena Oliveri[1], Reinhard Arnou[2], Ilaria Durosini[1], Paolo Guiddi[1], Evelyne Louis[3], Marie Vandevelde[3], Kristiaan Nackaerts[3], Meredith Y. Smith[4], Giulia Galli[5], Filippo de Marinis[6], Letizia Gianoncelli[6], Gabriella Pravettoni[1], Isabelle Huys**[2]**

Appendix SA: List of examples used in the focus group discussions

Potential positive effects	Explanation
Progression Free Survival (PFS)	PFS is the length of time during or after lung cancer treatment that a patient lives with the disease but it does not get worse.
Overall Survival (OS)	OS is the length of time (from diagnosis or treatment) that a patient is alive.
Overall Survival Rate	Survival rate is the percentage of people in a treatment group who are alive at a specific time point. ex. the five-year survival rate, would be the percent of lung cancer patients alive 5 years after starting the treatment.
Disease size (defined as "Response Rate", RR)	Response rate is the percentage of patients whose lung cancer shrinks or disappears after treatment.
Incidence of adverse events (tolerability)	The number of newly diagnosed patients each year who experience new unfavorable symptoms or events during treatment.
Increase in quality of life	Improvement in quality of life over a specific time period. Quality of life is the overall enjoyment of life.
Time to deterioration of specific symptoms (e.g. cough, chest pain, dyspnea)	The time until a symptom worsens (by a specific amount).
**Potential negative effects**	**Explanation**
Hypothyroidism	Too little thyroid hormone in blood. This can cause a weight gain, constipation (the stool becomes hard, dry, and difficult to pass), dry skin, and sensitivity to the cold. Also called underactive thyroid.

Hyperthyroidism	Too much thyroid hormone in blood. This can cause weight loss, chest pain, cramps, diarrhea, and nervousness. Also called overactive thyroid.
Pneumonia	A severe inflammation of the lungs.The main function of the lungs is breathing. If the lungs do not work properly, the ability of lungs to breathe is reduced. Pneumonia may cause a decrease in the amount of oxygen that blood can absorb from air breathed into the lung. This can manifest as shortness of breath, fever, cough with or without phlegm.
Reduced red blood cell count	A condition in which the number of red blood cells in your blood is below normal. Red blood cells carry fresh oxygen throughout the body. When the number of red blood cells is decreased, you can feel tired and weak and look pale. Usually this condition is discovered when a blood test is performed.
Decreased appetite	A decreased desire to eat, making you lose weight.
Infusion reaction	A type of hypersensitivity reaction that develops during or shortly after administration of a drug. You may have itch, have red areas on the skin, fever, feel cold with shivering and a rise in temperature, often with a lot of sweating and chills. Severe cases can lead to bronchospasms (when the muscles in the lungs tighten, causing restricted airflow) and cardiovascular collapse (a sudden loss of blood flow to the brain and other organs, causing altered mental status and low blood pressure). This is an unpredictable event which can appear with any new drug.
Renal failure	A condition in which the kidneys stop working and are not able to remove waste and extra water from the blood or keep body chemicals in balance. Acute or severe renal failure happens suddenly (for example, after an injury) and may be treated and cured. Chronic renal failure develops over months to years, and may be caused by conditions like high blood pressure or diabetes, and cannot be cured. Chronic renal failure may lead to total and long-lasting renal failure, called end-stage renal disease (ESRD). A person in ESRD needs dialysis (the process of cleaning the blood by passing it through a membrane or filter) or a kidney transplant. Also called kidney failure.
Myositis	Any condition causing inflammation in muscles. Weakness, swelling, and pain are the most common myositis signs. Sometimes there are no symptoms at all and the condition is discovered at blood test. For most people symptoms will be mild and won't last long. In rare cases, there can be some serious complications. In the most severe cases, myositis can affect the muscles that carry out tasks such as breathing and swallowing requiring specific support for ventilation and nutrition.

Colitis	Inflammation of the colon. Symptoms of colitis depend upon the cause and may include abdominal pain, cramping, diarrhea, with or without blood in the stool (one of the hallmark symptoms of colitis).
Nausea	A feeling of sickness or discomfort in the stomach that may come with an urge to vomit.
Reduced white blood cell count	A condition in which there is a lower-than-normal number of white blood cells in the blood. White blood cells protect you against infectious diseases. If levels of white blood cells are lower than usual, this may be a sign that the person has reduced capacity to fight infections, being prone to develop severe infections also for otherwise common and not dangerous agents (e.g. influenza virus, some bacteria). The most common manifestation of reduced white blood cell count is fever. In the other cases this is a condition which is discovered incidentally at blood test.
Edema	Swelling caused by excess fluid in body tissues.
Thrombocytopenia	A condition in which there is a lower-than-normal number of platelets (a type of blood cells) in the blood. The function of platelets is to take part in the process of wound healing and stopping blood loss in case of trauma. Therefore, thrombocytopenia may result in easy bruising and excessive bleeding from wounds or spontaneous (that is, in absence of trauma) bleeding in mucous membranes and other tissues. Sometimes this condition is incidentally discovered with a blood test.

Appendix SB
Preference-sensitive areas according to the US Food and Drug Administration

The FDA for the evaluation of medical devices defined *"Parameters for Identifying Priority Patient Preference-Sensitive Regulatory Areas"*: i) FDA staff are looking to better understand the full impact of the disease or condition and treatment options on patients and/or caregivers; ii) patients may value the benefits and risks of a technology or treatment differently from healthcare professionals and/or caregivers; iii) population-level differences in patient perspectives are not well understood because of differences in: demographic characteristics, stages of a disease, disease phenotype and iv) there is significant public health impact (such as high mortality or morbidity rates and high prevalence rates of the disease or few treatment options available such as in rare diseases). The FDA used these to develop a priority list of *"patient preference-sensitive areas"* in different disease areas. In oncology (see https://www.fda.gov/about-fda/cdrh-patient-engagement/priority-list-patient-preference-sensitive-areas#oncology), the FDA formulated the following preference-sensitive areas which we attempt to apply to our study, although with the complexity that these areas were identified in the medical device setting whereas our study focuses on drugs:

- **Patient Values in Diagnosis and Treatment: Incremental increase in survival vs. risk of *device*-related toxicity.** This is true in the context of advanced stages (stage III/IV) of NSCLC as here recently, an increasing amount of treatment options and treatment combinations have become available (e.g. therapeutic approach for oligometastatic NSCLC with the possibility to combine chemotherapy, immunotherapy, local stereotactic body radiotherapy (SBRT). These treatments are associated with different benefits (PFS, OS, response rates) and risks.
- **Patient Values in Diagnosis and Treatment: Oncology device treatments: quality of life vs. survival.** This is also true in the context of advanced stages (stage III/IV) of NSCLC as the above mentioned treatments could cause possible physical changes (e.g. weight increase, bleeding and hair loss) and side-effects (e.g. pain, nausea and vomiting, breathing problems and fatigue), that differently affect body image perception (Bahrami et al., 2017) and quality of life (Blinman et al, 2010, Grassi et. al, 2017). Furthermore, from a psychological point of view, patients' cancer response could be influenced by declines in performance status and functional activity, poor concentration, rumination, memory impairment, altered sexuality and the instability of one's own emotional status (e.g. fears, anxieties, worries, and sadness). The clinical decision process can be influenced not only by the variety of treatment options available and the different variables but also by the psychological impact that a given treatment can have (Hajjaj et al., 2010; Blinman et al, 2010). Therefore, decision-makers need to consider the added value of treatments with a stronger impact on their quality of life (chemo-immunotherapy) than alternatives that may be less effective but with fewer adverse events and a lower reduction in quality of life (immunotherapy).
- **Impact of Uncertainty in Benefit-Risk Tradeoffs: Uncertainty related to benefits of oncology device treatments vs. unknown risk and/or high risk.** There are no guidelines for clinicians and patients' decisions between the above two treatments and in the absence of clear comparisons between possibilities and best choice (Shafique & Tanvetyanon, 2019; von Dincklage et al., 2013), schedules and toxicity become two crucial elements guiding health-care decision-making regarding personalized therapeutic approach. Moreover, experts recommend to discuss treatment options with patients and individualize the choice (Novello et al., 2016). Consequently, the consideration of patients' preferences about benefits, risks and other treatment outcomes, as evidenced in Clinical Practice Guidelines of the European Oncology Society (ESMO, 2016), is essential in order to classify the decision as a "preference-sensitive decision".

Appendix SC
3.1 Focus group general guidelines

1. During recruitment
- The focus group will involve 6 participants
- The following inclusion criteria will be applied to patients:
    - Adults (≥ 18)
    - Histological or cytological diagnosis of NSCLC stage III or IV as classified by the UICC TNM VIII Edition;
- The following exclusion criteria will be applied to patients:
    - Cognitive impairment or inadequate verbal skills that may render them incapable of informed consent (as evaluated by the clinician);
    - Inability to understand study materials (as evaluated by the clinician);
    - Physical or psychological impairment that prohibits their participation in an interview or focus group (as evaluated by the clinician);
- Book a venue/meeting room that is neutral and easily accessible to patients and that allows for a circle seating around a table
- Provide the participants with instructions to get to the venue and room
- Provide your/assistants' phone number(s) to the participants
- Print the information sheet, informed consents and surveys[1]

2. Day of the focus group
- Have enough information sheets, consent forms and surveys with you
- Seating arrangements: circle seating around table
- Provide writing material and paper
- Provide name cards (mentioning only the first name of participants) to put on the table
- Provide water/coffee/tea + something to eat
- If slides are available, set up slide show
- Take two audio recorders (+ extra batteries) with you and place these on the table at opposite sides
- Dress code for moderator and assistant(s): casual, pants, and blouse is OK, no suits, no suit jackets/blazers, not too formal

3. Moderator and assistant team

3.1 Attitude of moderator:
- Exercise mild unobtrusive control (moderate the discussion but do not interrupt too often)
- Adequate knowledge of topic
- Appears like the participants
- Use purposeful small talk
- Alert and free from distractions (put sound of phone off and do not have your phone on you)
- Have the discipline of listening and apply active listening:
    - Verbal reactions:
        - Short verbal responses (e.g. "I see", "Yes", "Ok", avoid "that's good", "excellent")
        - Use pauses and probes
            - 5 second pause probes: "Would you explain further?" "Would you give an example?"
        - Listen for inconsistent/vague/cryptic comments and probe for understanding, e.g. "I don't understand."

---

[1] *Including questions about general characteristics, clinical characteristics and health literacy questions (Chew et al., 2008), see appendix G.*

        - Consider asking a final yes/no question
        - Uses probes to refocus the discussion when the discussion goes off-topic, e.g. “Now that we have talked about XXX, I would go back/address the question/topic…”)
    - Nonverbal reactions:
        - Head nodding
- Familiar with questioning route (know this protocol very well)
- Take into account the different types of participants and try to balance the conversation while addressing the obligatory topics: dominant talkers, shy participants, etc.

3.2 Tasks of the assistant:

- Handles logistics (location of refreshments, bathrooms, emergency exits)
- Collects consent forms and surveys
- Takes careful notes on paper or on laptop:
    - Anticipate that others will use your notes. Notes sometimes are interpreted days or weeks following the focus group when memory has faded. Consistency and clarity are essential.
    - It is essential that this information is easily identified and organized. The notes should be divided according to the focus group questions/topics and include time indications
    - Your notes will contain different types of information:
        - Quotes:
            - Listen for notable quotes, the well said statements that illustrate an important point of view. Listen for sentences or phrases that are particularly enlightening or eloquently express a particular point of view. Place name or initials of speaker after the quotations. Usually, it is impossible to capture the entire quote. Capture as much as you can with attention to the key phrases. Use three periods ... to indicate that part of the quote was missing.
        - Key points and themes for each question
            - Typically, participants will talk about several key points in response to each question. These points are often identified by several different participants. Sometimes they are said only once but in a manner that deserves attention. At the end of the focus group the assistant moderator will share these themes with participants for confirmation.
        - Follow-up questions that could be asked
            - Sometimes the moderator may not follow-up on an important point or seek an example of a vague but critical point. The assistant moderator may wish to follow-up with these questions at the end of the focus group.
        - Big ideas, hunches, or thoughts of the recorder
            - Occasionally the assistant moderator will discover a new concept. A light will go on and something will make sense when before it did not. These insights are helpful in later analysis.
        - Other factors
            - Make note of factors which might aid analysis such as passionate comments, body language, or non-verbal activity. Watch for head nods, physical excitement, eye contact between certain participants, or other clues that would indicate level of agreement, support, or interest. Nonverbal: movements, attitudes, emotions. These will be

indicated in the notes by using a different color our by highlighting the text describing nonverbal reactions
        - These notes will be used by the assistant at the end of the focus group discussion to summarize the focus group (see end guideline)
- Controls for equal participation by all participants and informs the moderator if some participants are not getting the chance to participate
- Monitors audio recording equipment
- Time management via discrete signs to the moderator
- Gives a general summary based upon the above mentioned notes at the end of the focus group. The summary should encompass the different questions/topics addressed in the discussion and give a balanced view of the different opinions expressed

3.2 Focus group discussion guide

Everything below in *italic* is to be said to the participants, everything in black is guidance and can be told to the participants in your own words. In **bold** an indication of timing of actions is given; however, this is an indication of time, it is more important to finish the topics than to rush through the focus group.

00:00 Welcome the participants while they arrive
- Create warm and friendly environment
    - Provide coffee and tea
    - Interact with participants, and stimulate interaction between participants
- Make circular seating arrangements for participants according to their needs
- Provide the information sheet and let them fill in the consent form and survey

00:10 Check whether all participants have arrived. If not, the assistant will try to reach these persons via telephone, in a separate room. If the missing participants cannot be reached, the focus group will start without these persons. Let the arrived participants fill in the necessary forms and start the focus group with a general introduction:
- *Welcome, my name is* (your first name) *and I will be your moderator today. In addition, I brought* (first name of assistant) *to help me with the focus group*
- *My role as moderator will be to guide the discussion*
- *The discussion that we will have today is about what you think about medical treatments for lung cancer and what you expect from lung cancer treatments. We want to learn from you what you would value in a lung cancer treatment, what would make that you take or not take a lung cancer treatment and why*
- *We want to have this discussion with you as patient, since you are the eventual user of the treatment*
- *The opinions collected today will be used to develop a survey that we will spread out to a large number of lung cancer patients. This will allow us to quantify (put in numbers) the opinions we collect today*
- *The opinions collected today will be not be used to change anything about your ongoing or future treatment*
- *In the end, we hope that the opinions we collect in this research will be used by pharmaceutical companies to develop lung cancer treatments tailored to patient needs, and by health authorities to make decisions on whether a specific lung cancer treatment can become available on the market or whether it can be reimbursed*
- *This study is part of a large European project called PREFER. This research project looks at how and when patient preferences for new treatments should be incorporated into the*

drug development process. PREFER aims to make the development of drugs and decisions about drugs more patient-centered.

- *The focus group will take about 1 hour and a half*
- *There will be a break in the middle of the discussion of 10 minutes*

00:12 Explain the "rules":

- *There are no right or wrong answers, only differing points of view*
- *We are looking for your opinions and hope for a nice discussion*
- *It is possible that you do not agree with all opinions, but it would be nice if you could listen respectfully to each other*
- *Since this is an informal discussion, we will address each other only by their first name as indicated on the name cards*
- *We ask you to turn off the sound of your phones*
- *If there are any questions or terms that are used during the focus groups that are not clear to you, please let us know*
- *To be able to fully focus on the focus group as moderator and assistant, we will audio-record our conversation today. Only researchers on the project will have access to the recordings. These recordings will be eventually destroyed after being transcribed into a computer file when identifiable data like names and date of birth will be deleted and your responses will be recognized just by a code. More details regarding the data coding and processing appear on your information sheet. If you do not want that your opinions are being audio recorded, you should not sign the information sheet nor choose to participate in the focus group*
- *To be able to fully understand what everybody says and also to help our analysis, it would be very helpful for the analysis if only one person is speaking at a time*
- *Are there any questions about what I just mentioned to you?*
- *We will now start the recording. Is that OK for everybody?*

00:15 *To get to know each other, we would like to do a round-the-table where each of us tells us shortly* (max 1min each) *a little bit more about themselves (name, background) and why you decided to join today's discussion.*

00:30 *As mentioned in the beginning, the discussion that we will have today is about what you think about medical treatments for lung cancer and what you expect from lung cancer treatments. We want to learn from you what you would value in a lung cancer treatment, what would make that you take or not take a lung cancer treatment and why.*

Therefore, we would like to know: when you undergo a treatment for lung cancer, what type of improvement do you expect from it? With improvement we mean benefits or desirable effects.

- *Why?*

Some examples of improvements could be (see Appendix B)

- *Are there any other types of improvements you can think of?*
- *Why are these important to you?*

00:45 Break. *We will now have a break of 10 minutes. Please feel free to use the restroom, take water/coffee/tea/something to eat.*

00:55 *Until now we discussed what benefits you expect from lung cancer treatments. Lung cancer treatments may also be associated with side-effects. With side effect we mean risks or undesirable effects of the treatment. When you undergo a treatment for lung cancer, what type of side-effects would make you want to doubt whether you want to keep on taking the treatment?*

- *Why?*

What type of these side-effects would make you stop taking the treatment?

- *Why?*

Some examples of side-effects could be (See Appendix B)

- *Are there any other types of side-effects you can think of?*
- *Why are these important to you?*

01:10 *We have now discussed types of improvements and side-effects that would influence your choice to take a lung cancer treatment. Thinking about the improvements and side-effects we just discussed:*

- *What type of side-effects would make you want to reconsider whether you want to continue the treatment?*
- *What type of improvements would make you want to accept more of the side-effects we just talked about?*

01:25 *Are there any other aspects of lung cancer treatment, besides the different side-effects and improvements we just talked about that would influence your choice to take or stop taking a lung cancer treatment? (open question)*

- *Why?*

Some examples of other treatment characteristics could be (see Appendix B)
- *Are there any other treatment characteristics you can think of?*
- *Why are these important to you?*

01:40 Finish. *The focus group is now finished*
- Summarize the lessons learnt of today's discussion – by assistant
- Ask if the summary is correct, or if you have forgotten something
- Ask if there are any questions
- Thank all participants for their participation

Appendix SD: Answer sheet completed by all participants

Dear participant,

Please fill out the information below. We would like to collect your answers on the questions below to learn more about you as a focus group participant. The answers of all participants on these questions (excluding the names of participants) will be summarized as group characteristics in reports and publications on this focus group. All information provided by you will be anonymized during the analysis, meaning the results of this study will never be linked to your name and identity. If a question is not clear to you, please ask the focus group moderator or an assistant for more information.

Section 1: Personal characteristics

1. What is your name (first name and family name)?
2. What is your age?
3. To which gender identity do you most identify?
    a. Female
    b. Male
    c. Other
    d. Prefer not to answer
4. What is the highest level of education that you completed?
    a. I did not complete high school
    b. High school
    c. Bachelor's degree
    d. Master's degree
    e. Ph.D.
5. Which country or countries are you a citizen of?
    a. Belgium
    b. Italy
    c. Other, please specify:

Section 2: Experiences as a patient

1. At what age were you diagnosed with lung cancer?
2. Are you currently taking any medication specific for lung cancer? ***(yes/no)***
3. If you are currently taking medication, what type of medication are you currently taking?
4. How many treatment lines have you received (the current one included)? One treatment line of treatment refers to one drug or a combination of drugs that are usually given for a specific time duration
5. If you are not currently taking medication, what is the reason for this?
6. How often do you have someone (like a family member, friend, hospital/clinic worker, or caregiver) help you read hospital materials?
    a. Always
    b. Often
    c. Sometimes

d. Occasionally
e. Never

7. How often do you have problems learning about your medical condition because of difficulty understanding written information?
    a. Always
    b. Often
    c. Sometimes
    d. Occasionally
    e. Never

8. How confident are you filling out medical forms by yourself?
    a. Extremely
    b. Quite a bit
    c. Somewhat
    d. A little bit
    e. Not at all

Thank you for completing this short survey!
You can now hand this completed survey to your focus group moderator.
